# Assessing thyroid peroxidase antibodies in Emirati medical students: a cross-sectional pilot study

**DOI:** 10.3389/fendo.2025.1582933

**Published:** 2025-08-25

**Authors:** Afrin Pathan, Javed Yasin, Charu Sharma, Juma Alkaabi, Adnan Agha

**Affiliations:** ^1^ Department of Internal Medicine, College of Medicine and Health Sciences, United Arab Emirates University, Al Ain, United Arab Emirates; ^2^ Department of Genetics and Genomics, College of Medicine and Health Sciences, United Arab Emirates University, Al Ain, United Arab Emirates

**Keywords:** autoimmune thyroid disease, Hashimoto’s thyroiditis, thyroid peroxidase antibody, hypothyroidism, United Arab Emirates

## Abstract

**Introduction:**

Anti-thyroid peroxidase antibodies (TPO-Ab) are detectable in almost all patients with autoimmune thyroid disease or Hashimoto’s thyroiditis (HT) but may also be present in healthy individuals. HT affects women to a greater extent than men and can lead to overt hypothyroidism, which may increase the risk of miscarriage. There are no local data available on the prevalence of TPO-Ab among healthy women in the United Arab Emirates. The objective of this study was to determine the prevalence of TPO-Ab and assess thyroid function in healthy medical students.

**Methods:**

This cross-sectional study used convenience sampling to recruit participants without any history of medical illness or prescribed medications from the College of Medicine at United Arab Emirates University, after obtaining informed consent. Routine demographic, anthropometric, and biochemical data—including TPO-Ab and thyroid-stimulating hormone (TSH) levels—were collected. The normal reference ranges for TPO-Ab and TSH (according to kit-specific recommendations) were 0–34 IU/mL and 0.27–4.20 mIU/L, respectively.

**Results:**

A total of 90 healthy participants were enrolled (mean age: 19.83 ± 1.41 years), and all completed blood testing. All male participants (n = 27) had normal TPO-Ab levels, whereas eight female participants (12.7%; n = 63) had elevated TPO-Ab levels (mean: 40.03 ± 102.00 IU/mL). Three female participants (4.5%, n = 63) had elevated TSH levels without any clinical symptoms.

**Conclusion:**

Elevated TPO-Ab levels were observed in 12.7% (95% CI: 5.7%–23.5%) of young female participants. These preliminary findings suggest the need for larger prospective studies to evaluate the clinical significance of elevated TPO-Ab and possible related complications.

## 1 Introduction

Hashimoto’s thyroiditis (HT), also known as autoimmune thyroid disease, was first described in 1912 by Hakaru Hashimoto. It can cause cellular damage through sensitized T lymphocytes and/or autoantibodies binding to thyroid cell membrane receptors, leading to altered thyroid gland function (1). HT is one of the most common autoimmune diseases, affecting approximately 2% of women with overt clinical symptoms—10 times higher than the 0.2% prevalence in men. Subclinical disease affects an estimated 20% of women, with an incidence of 3–6/10,000 per year and a varied geographical distribution, ranging from 5.8% in Asia to 14.2% in Africa (2, 3).

Thyroid peroxidase (TPO), discovered more than four decades ago, is a thyroid microsomal antigen. It is a 107-kDa membrane-associated hemoglycoprotein that catalyzes the iodination and coupling of tyrosine residues to synthesize thyroid hormones (organification) (4, 5). Antibodies to thyroid peroxidase (TPO-Ab), measured using sensitive and standardized immunoassays, are elevated in more than 95% of patients with HT and nearly 85% of patients with Graves’ disease (6, 7).

Several previous studies have reported the prevalence and distribution of TPO-Ab in large cohorts of euthyroid individuals. These studies emphasized the variability in TPO-Ab levels across populations and the need for careful calculation of reference intervals to accurately interpret TPO-Ab measurements (8). Factors such as iodine intake, genetic predisposition, and environmental influences are crucial for understanding and managing thyroid autoimmunity (9). A large-scale European study reported elevated TPO-Ab levels in 13.9% of women and 2.8% of men without thyroid disease (10). Similarly, data of another large cohort from the National Health and Nutrition Examination Survey (NHANES III) reported elevated levels of thyroid stimulating hormone (TSH), and a higher prevalence of anti-thyroid antibodies in women compared with men, and in whites and Mexican Americans compared with Blacks. Antibody prevalence also increased with age (8, 11). Studies from Africa have reported TPO-Ab prevalence rates in normal adult populations ranging from 2.6% to 7% (12, 13). A study in Isfahan, Iran reported that 35.8% of healthy women tested positive for TPO-Ab following iodine supplementation, suggesting a potential link between iodine intake and increased thyroid autoimmunity (14). A previous study indicated that the prevalence of TPO-Ab is between 5% and 20% among women of childbearing age (15), and higher in women with subfertility—ranging from 10% to 31% in those with a history of recurrent pregnancy loss (17%–33%) compared with 6% to 20% in the general population (16). In Saudi Arabia, TPO-Ab prevalence was reported at 11% in adults and slightly higher, at 14%, among school-aged children in the normal population (17, 18). Environmental factors such as dietary habits, psychological stress, and exposure to environmental toxins have also been shown to affect thyroid health and autoimmunity (9, 19).

To the best of our knowledge, no studies have assessed the prevalence of TPO-Ab in the general population of the United Arab Emirates (UAE), despite global studies showing a significant presence of these antibodies in various populations. Therefore, the aim of this study was to determine the prevalence of TPO-Ab and assess thyroid dysfunction among healthy medical students at the United Arab Emirates University (UAEU).

## 2 Participants and methods

### 2.1 Participant selection

This cross-sectional pilot study used convenience sampling to recruit participants from the College of Medicine and Health Sciences (CMHS) at the United Arab Emirates University (UAEU) between March and June 2024. The study was approved by the United Arab Emirates University Human Medical Research Ethics Committee (UAEU.HREC) (approval number: ERH_2023_2353).

A total of 112 students were invited through announcements in classes and notices, and 90 students agreed to participate (response rate: 80.4%). There were no participant dropouts or missing data. An on-site camp was established at the medical college to facilitate participation.

The selection of this particular population was based on its representation of the young, educated Emirati demographic, as the CMHS is the only public medical college in the UAE that admits students from all seven Emirates. Furthermore, all participants were from medical backgrounds, which enabled them to provide detailed information regarding thyroid-related illnesses in their personal or family medical histories. Informed consent was obtained from all participants.

Eligible participants were medical students without any known health problems and who were not taking any medications or herbal remedies. Iodine supplement use was ruled out through direct questioning during the interview, although the limitations of self-reporting are acknowledged. Students with a family history of known thyroid disease were excluded.

Demographic, anthropometric, and smoking data were collected. No personally identifiable information was recorded, and participant confidentiality was maintained throughout the study.

### 2.2 Sample size calculation

Using an estimated TPO-Ab prevalence of 15%, based on regional studies (14, 17), and assuming 80% power and a significance level (α) of 0.05, we calculated that a minimum of 60 participants would be needed to detect this prevalence with adequate precision (power 80%, alpha level 0.05, one-sample proportion test.

### 2.3 Blood sampling

After an overnight fast, blood samples were collected from participants via venipuncture. Plasma was separated and stored at –20°C until analysis. Samples were processed for biochemical analyses, including low-density lipoprotein (LDL), high-density lipoprotein (HDL), triglycerides, total cholesterol, and serum creatinine. Lipid profile and creatinine were measured as part of a comprehensive health assessment to exclude participants with undiagnosed metabolic or renal conditions that could potentially impact the interpretation of thyroid function tests or TPO-Ab levels.

Circulating levels of total cholesterol, triglycerides, HDL, LDL, and serum creatinine were measured using the automated analyzer Integra 400 Plus (Roche Diagnostics, Mannheim, Germany). TSH, total thyroxine (T4), and TPO-Ab levels were measured using the automated cobas e411 analyzer (Roche Diagnostics, Mannheim, Germany). Elecsys T4 (reference: 09007741190), Elecsys TSH (reference: 08429324190), and Elecsys anti-TPO (reference: 06368590190) were measured using chemiluminescent immunoassays. The cobas e411 is an automated, random-access, multichannel analyzer designed for quantitative and qualitative *in vitro* determination of analytes using electrochemiluminescence (ECL) technology.

Normal reference ranges for TPO-Ab, TSH, and total serum T4 were 0–34 IU/mL, 0.27–4.20 mIU/L, and 66.0–181.0 nmol/L, respectively, as per kit-specific recommendations.

### 2.4 Statistical analysis

Statistical analyses were performed using SPSS version 29.0 (IBM Corp., Armonk, NY, USA). Continuous variables were tested for normality using the Shapiro–Wilk test. Data are presented as mean ± standard deviation (SD) for normally distributed variables and as median (interquartile range) for non-normally distributed variables. Categorical variables are presented as frequencies and percentages, with 95% confidence intervals (CI) calculated using the Wilson method.

Fisher’s exact test was used to examine the association between elevated TPO-Ab and abnormal TSH levels. Due to the small sample size and low event rates, multivariate analysis was not performed; however, body mass index (BMI), sex, and smoking status were examined descriptively for any apparent associations with TPO-Ab levels. All tests were two-tailed, with p < 0.05 considered statistically significant. Exact p-values are reported where appropriate. The researchers involved in analyzing the data were blinded to the demographics to avoid bias.

## 3 Results

A total of 90 medical students, including 63 women (70%), participated in this study. All participants were aged 18–24 years (mean ± SD: 19.83 ± 1.41 years), which falls within the definition of the late adolescent age group (20). These students represented all seven Emirates of the UAE, with the highest number from Al Ain (n = 28; 31.1%) and Abu Dhabi (n = 26; 28.9%), and the lowest (n = 1; 1.1% each) from Ajman and Umm Al Quwain. None of the participants had any current medical diagnosis, were on prescribed medication, or had a personal or family history of thyroid disorders. Only one student had a history of smoking. A total of 14 participants (15.5%) had a BMI higher than 29.9 kg/m^2^. None of the participants showed clinical signs or symptoms of hypothyroidism or hyperthyroidism.

All participants completed the biochemical analyses. The mean ± SD values for total serum T4, TSH, and TPO-Ab were 120.82 ± 20.17 nmol/L, 1.85 ± 1.44 mIU/L, and 32.29 ± 85.98 IU/mL, respectively. **Table 1** shows the clinical and biochemical characteristics of all participants.

**Table 1:** Clinical and biochemical characteristics of all participants (n = 90).

Characteristics	Mean	± Standard deviation
Age (years)	19.83	1.41
Sex	63 females (70%)	
Body mass index (kg/m^2^)	25.31	6.08
Creatinine (μmol/L)	56.61	9.40
Urea (mmol/L)	2.54	0.66
Alanine transaminase (U/L)	15.08	3.95
Aspartate transaminase (U/L)	20.72	4.09
Albumin (g/L)	51.63	4.09
Fasting glucose (mmol/L)	5.35	0.61
Total cholesterol (mmol/L)	4.80	0.72
High-density lipoprotein (mmol/L)	1.84	0.33
Low-density lipoprotein (mmol/L)	2.89	0.68
Triglyceride (mmol/L)	0.85	0.28
Total thyroxine, T4 (nmol/L)	120.82	20.17
Thyroid stimulating hormone, TSH (mIU/L)	1.85	1.44
Thyroid peroxidase antibody, TPO-Ab (IU/mL)	32.29	85.98

**Figure 1:**
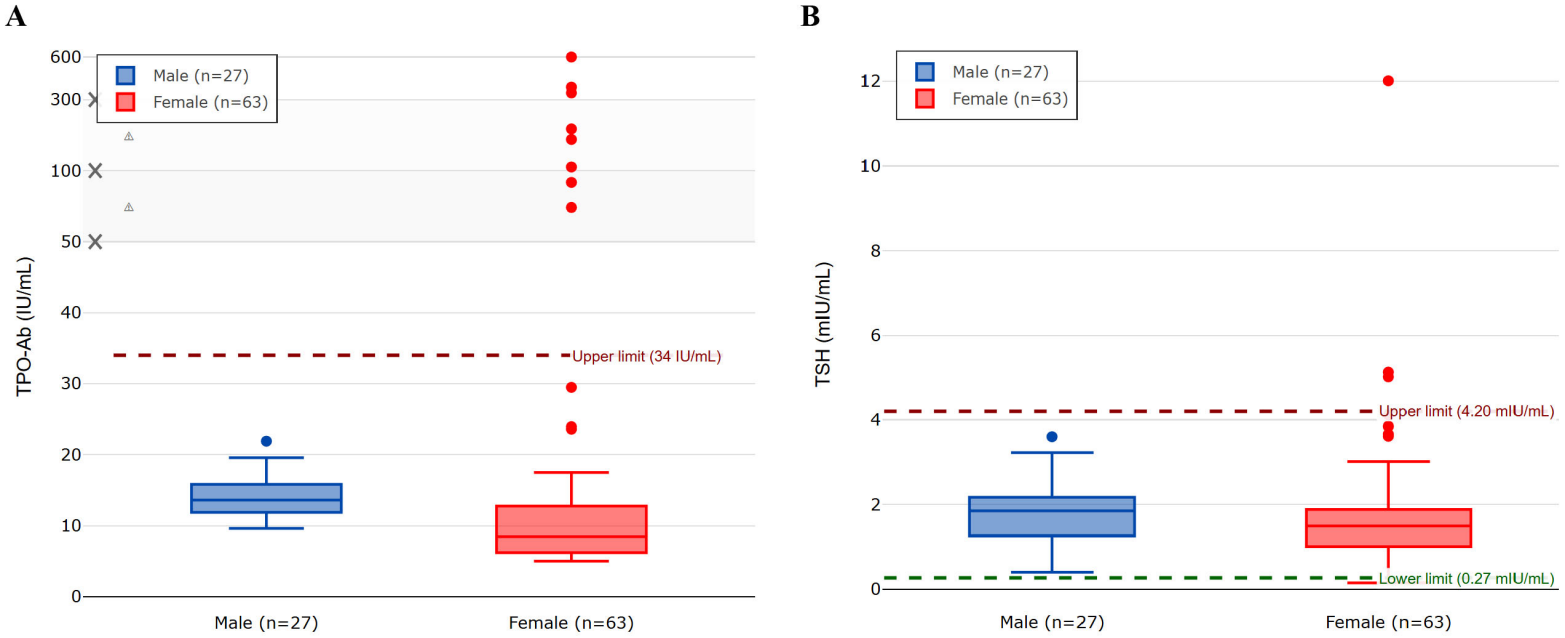
The figure displays box plots for **(A)** TPO-Ab levels and **(B)** TSH levels in male (n=27) and female (n=63) participants. The central line in each box indicates the median value, the box boundaries represent the interquartile range (IQR; 25th to 75th percentile), and the whiskers extend to 1.5 times the IQR from the box boundaries. Circles represent individual outlier values that fall beyond the whiskers. Panel **(A)** uses a non-linear y-axis scale (0-50 linear, then compressed) to better visualize both the main data distribution and outliers. The normal reference ranges are 0–34 IU/mL for TPO-Ab and 0.27–4.20 mIU/mL for TSH.

All male students (n = 27) had TSH levels within the normal range, and none showed elevated TPO-Ab levels. Further analysis of the female participants (n = 63) revealed a mean age of 19.7 ± 1.51 years and a mean BMI of 24.23 ± 5.02 kg/m^2^. Only one female student had a BMI ≥ 30 kg/m²; her TPO-Ab level was within the normal range. **Table 2** shows the biochemical characteristics of female participants. **Figure 1** shows the distribution of TPO-Ab and TSH levels by gender, demonstrating that elevated TPO-Ab levels were observed exclusively among female participants.

**Table 2:** Biochemical characteristics of female participants (n = 63).

Characteristics	Mean	± Standard deviation
Creatinine (μmol/L)	56.60	9.48
Total cholesterol (mmol/L)	4.80	0.71
High-density lipoprotein (mmol/L)	1.84	0.33
Low-density lipoprotein (mmol/L)	2.89	0.68
Triglyceride (mmol/L)	0.85	0.28
Total thyroxine, T4 (nmol/L)	116.34	17.13
Thyroid stimulating hormone, TSH (mIU/L)	1.83	1.65
Abnormal TSH (>4.20 mIU/L)	3 (3.3%)	
Thyroid peroxidase antibody, TPO-Ab (IU/mL)	40.03	102.01
Abnormal TPO-Ab (>34 IU/mL)	8 (12.7%)	

Eight female participants (12.7%, 95% CI: 5.7%–23.5%; n = 63) had elevated TPO-Ab levels, with a mean of 40.03 ± 102.00 IU/mL. The standard deviation in TPO-Ab levels (85.98 IU/mL overall, 102.00 IU/mL in women) was high, which reflects the presence of outliers, including one participant with a TPO-Ab level of 600.0 IU/mL. The distribution of TPO-Ab values was non-normal, as confirmed by the Shapiro–Wilk test (p < 0.001), which is typical for antibody measurements in population studies. Sub-analysis of these eight participants revealed TPO-Ab levels ranging from 72.0 to 600.0 IU/mL (normal range: 0–34 IU/mL). Elevated TSH levels (>4.20 mIU/L) were observed in three female participants (4.8%, 95% CI: 1.0%–13.3%; n = 63), including one individual with a markedly elevated TSH of 12.0 mIU/L and normal total T4 levels, without clinical signs of hypothyroidism. Fisher’s exact test was used to correlate the presence of elevated TPO-Ab in predicting abnormally increased TSH among these eight (n = 63) female participants with raised TPO-Ab, which revealed a p-value of < 0.001, indicating a significant correlation.

## 4 Discussion

Our study showed increased TPO-Ab levels in 12.7% of Emirati female participants, representing the late adolescent age group in the UAE. This demographic marks the start of the childbearing years for women, who may face an increased risk of miscarriage if they later develop hypothyroidism. None of the male participants in our study had abnormal TPO-Ab levels. Based on *post hoc* power analysis, the observed TPO-Ab prevalence of 12.7% in young Emirati women represents, to our knowledge, the first reported estimate of its kind in the UAE.

A retrospective study of 366 UAE nationals attending thyroid clinics found that 220 (60%) participants had abnormal TPO-Ab and/or thyroglobulin antibody (TgAb) levels. Among these, 188 (85.5%) were women aged 31–40 years. Of those 188 women, 42% had euthyroid, 42% had hypothyroidism, 7% had hyperthyroidism, 5% had subclinical hypothyroidism, and 2% had subclinical hyperthyroidism (21). This study also identified obesity (65%) as the most common modifiable comorbidity and a family history of thyroid disease in first-degree relatives (37%) as a significant nonmodifiable risk factor. The high rate of consanguineous marriages in the UAE (21) has been cited as a possible explanation for the elevated familial risk. In contrast, nearly all participants in our study were non-obese, and the one participant with obesity had normal TPO-Ab levels.

This study showed the prevalence of elevated TPO-Ab levels among young, apparently healthy Emirati women without clinical signs of thyroid dysfunction. There are currently no UAE-specific data on population-based reference ranges or TPO-Ab prevalence established using the 90th or 95th percentile. It is well documented that TPO-Ab positivity is prevalent among women and older individuals (8). Abnormal TPO-Ab levels can be associated with non-thyroidal conditions, including an increased risk of miscarriage (22). The relatively high prevalence observed in our young female cohort may increase further with age, potentially placing them at high risk for thyroidal and non-thyroidal illnesses. Currently, there is no medical program in the UAE that screens for TPO-Ab at least once in a lifetime to identify the risk of developing future thyroid dysfunction.

Findings from the longitudinal Tehran Thyroid Study (TTS) reported a TPO-Ab positivity among young, middle-aged, and elderly women from the general population with sufficient iodine intake. The study showed that the prevalence of TPO-Ab positivity in young women varies, with a prevalence of approximately 14.9% in this demographic (23). This is quite similar to our results with 12.7% of our female students having raised TPO-Ab.

A family history of thyroid disease can increase the likelihood of elevated TPO-Ab levels in young women. Therefore, screening for anti-thyroid antibodies may be justified in women of reproductive age, particularly those planning pregnancy, although the presence of TPO-Ab positivity in young women is not associated with overt thyroid dysfunction (24). All students in our study were interviewed, and those with family history of thyroid disease were excluded.

Elevated TPO-Ab levels during pregnancy have been linked to an increased risk of miscarriage and preterm delivery. Supplementation with levothyroxine in euthyroid women with TPO-Ab did not significantly increase the rate of live births compared to placebo. Identifying these antibodies can nonetheless allow for closer monitoring and timely intervention to optimize maternal and fetal outcomes (25). Screening for TPO-Ab may help detect autoimmune thyroid conditions, such as HT and Graves’ disease, even before clinical symptoms appear. This is particularly relevant for women, who are more susceptible to thyroid disorders due to hormonal and genetic factors (26). Our findings suggest that larger prospective studies are warranted to evaluate whether TPO-Ab screening might benefit specific high-risk populations, such as women of childbearing age.

There is currently no consensus on the treatment of subclinical hyperthyroidism, although anti-thyroid drugs or radioiodine therapy may be considered due to the long-term risks of atrial fibrillation and loss of bone density (27). Many individuals with TPO-Ab positivity may remain in a subclinical phase or asymptomatic throughout their lives (28). Therefore, while TPO-Ab positivity alone does not confirm the onset of thyroid dysfunction, it may serve as an early marker for the potential development of HT or Graves’ disease (29). The International Thyroid Association recommends annual monitoring of thyroid function in patients with increased TPO-Ab levels to enable early detection of overt hypothyroidism (30). In our study, three of the eight female students with elevated TPO-Ab (37.5%, 95% CI: 8.5%–75.5%) also had elevated TSH levels despite having no clinical features of hypothyroidism.

This pilot study has several important limitations. First, the cross-sectional study design prevents us from determining whether TPO-Ab positivity will lead to clinically significant thyroid dysfunction over time. Second, although we used convenience sampling from a single medical college that accepts Emirati students from all seven Emirates, the sample may not be fully representative of the broader population, limiting generalizability. Third, excluding participants with a family history of thyroid disease may have led to an underestimation of the true prevalence of TPO-Ab. Fourth, we did not perform thyroid ultrasound examinations, which could have identified morphological changes associated with autoimmune thyroid disease. Fifth, we did not measure thyroglobulin antibodies, vitamin D levels, selenium status, environmental toxin exposure, mental stress (e.g., anxiety/depression), or serum iodine levels—all of which are known to be associated with thyroid autoimmunity (9, 19). Finally, the relatively small sample size, particularly among male participants (n = 27), limits our ability to perform robust statistical comparisons or multivariate analyses.

Although the clinical significance of mildly elevated TPO-Ab levels remains poorly understood, this pilot study in a healthy young Emirati population helps to estimate the prevalence of elevated TPO-Ab levels. Elevated TPO-Ab levels should ideally be interpreted in conjunction with substantial functional or morphological implications, as it is not clear whether these elevations reflect a transient, reversible immune dysregulation or are indicative of an underlying, latent autoimmune thyroid condition. To address this gap, large-scale prospective clinical studies are essential to elucidate the risk of overt autoimmune thyroid diseases in this population. Such studies should focus on longitudinal assessments of thyroid function, autoantibody levels, and imaging findings to better understand the natural history and clinical impact of thyroid autoimmunity in this population.

## 5 Conclusion

This pilot study provides preliminary data on the prevalence of TPO-Ab in young Emirati female participants, with 12.7% testing positive for these antibodies. While these findings cannot be generalized to the broader population without larger confirmatory studies, they suggest that thyroid autoimmunity may be present in apparently healthy young Emirati women. Future prospective studies with larger and more representative samples are needed to determine the clinical significance of TPO-Ab positivity and to evaluate whether targeted screening in specific populations—such as females of reproductive age—might help identify individuals at risk for future complications, including thyroid dysfunction and fertility-related issues. Such studies should include longitudinal follow-up, thyroid imaging, and comprehensive assessment of biochemical, environmental, and psychological factors that may influence thyroid autoimmunity.

## Data Availability

The raw data supporting the conclusions of this article will be made available by the authors, without undue reservation.

## References

[1] Hashimoto H. Zur Kenntniss der lymphoatosen Veranderung der Schilddrüse (Struma lymphomatoa). Arch Klin Chirugie. (1912) 97:219–48.

[2] Akamizu T Amino A. Hashimoto’s thyroiditis. In: Feingold KR Anawalt B Blackman MR Boyce A Chrousos G Corpas E, editors. Endotext. MDText.com, Inc, South Dartmouth, MA (2000).

[3] Hu X Chen Y Shen Y Tian R Sheng Y Que H. Global prevalence and epidemiological trends of Hashimoto’s thyroiditis in adults: A systematic review and meta-analysis. Front Public Health. (2022) 10:1020709. doi: 10.3389/fpubh.2022.1020709, PMID: 36311599 PMC9608544

[4] Czarnocka B Ruf J Ferrand M Carayon P Lissitzky S. Purification of the human thyroid peroxidase and its identification as the microsomal antigen involved in autoimmune thyroid diseases. FEBS Lett. (1985) 190:147–52. doi: 10.1016/0014-5793(85)80446-4, PMID: 2995127

[5] Khoury EL Hammond L Bottazzo GF Doniach D. Presence of the organ-specific ‘microsomal’ autoantigen on the surface of human thyroid cells in culture: its involvement in complement-mediated cytotoxicity. Clin Exp Immunol. (1981) 45:316–28., PMID: 6172224 PMC1537363

[6] Feldt-Rasmussen U Høier-Madsen M Bech K Blichert-Toft M Bliddal H Date J. Anti-thyroid peroxidase antibodies in thyroid disorders and non-thyroid autoimmune diseases. Autoimmunity. (1991) 9:245–54. doi: 10.3109/08916939109007650, PMID: 1777557

[7] Weetman AP. Graves’ disease. N Engl J Med. (2000) 343:1236–48. doi: 10.1056/NEJM200010263431707, PMID: 11071676

[8] Zöphel K Saller B Wunderlich G Grüning T Koch R Wilde J. Autoantibodies to thyroperoxidase (TPOAb) in a large population of euthyroid subjects: implications for the definition of TPOAb reference intervals. Clin Lab. (2003) 49:591–600., PMID: 14651330

[9] Ferrari SM Fallahi P Antonelli A Benvenga S. Environmental issues in thyroid diseases. Front Endocrinol. (2017) 8:50. doi: 10.3389/fendo.2017.00050, PMID: 28373861 PMC5357628

[10] Bjoro T Holmen J Krüger O Midthjell K Hunstad K Schreiner T. Prevalence of thyroid disease, thyroid dysfunction and thyroid peroxidase antibodies in a large, unselected population. Health Study Nord-Trondelag (HUNT) Eur J Endocrinol. (2000) 143:639–47. doi: 10.1530/eje.0.1430639, PMID: 11078988

[11] Hollowell JG Staehling NW Flanders WD Hannon WH Gunter EW Spencer CA. T_4_, and thyroid antibodies in the United States population (1988 to 1994): National Health and Nutrition Examination Survey (NHANES III). J Clin Endocrinol Metab. (2002) 87:489–99. doi: 10.1210/jcem.87.2.8182, PMID: 11836274

[12] Okosieme OE Taylor RC Ohwovoriole AE Parkes AB Lazarus JH. Prevalence of thyroid antibodies in Nigerian patients. QJM. (2007) 100:107–12. doi: 10.1093/qjmed/hcl137, PMID: 17234716

[13] Johnson N Chatrani V Taylor-Christmas AK Choo-Kang E Smikle M Wright-Pascoe R. Population reference values and prevalence rates following universal screening for subclinical hypothyroidism during pregnancy of an Afro-Caribbean cohort. Eur Thyroid J. (2014) 3:234–9. doi: 10.1159/000367654, PMID: 25759799 PMC4311298

[14] Aminorroaya A Momenzadeh M Hovsepian S Haghighi S Amini M. WHO EMRO - Thyroid autoantibodies in women with and without thyroid disorders in an iodine-replete area. East Mediterr Health J. (2008) 14:325–32., PMID: 18561724

[15] Krassas GE Poppe K Glinoer D. Thyroid function and human reproductive health. Endocr Rev. (2010) 31:702–55. doi: 10.1210/er.2009-0041, PMID: 20573783

[16] Thangaratinam S Tan A Knox E Kilby MD Franklyn J Coomarasamy A. Association between thyroid autoantibodies and miscarriage and preterm birth: meta-analysis of evidence. BMJ. (2011) 342:d2616. doi: 10.1136/bmj.d2616, PMID: 21558126 PMC3089879

[17] Jammah AA Alshehri AS Alrakhis AA Alhedaithy AS Almadhi AM Alkwai HM. Characterization of thyroid function and antithyroid antibody tests among Saudis. Saudi Med J. (2015) 36:692–7. doi: 10.15537/smj.2015.6.11591, PMID: 25987111 PMC4454903

[18] Riad AS. Thyroid autoantibodies: High prevalence among schoolchildren and adolescents in Gizan, Saudi Arabia. Saudi Med J. (1995) 16:287–90.

[19] Benvenga S Antonelli A Vita R. Nutraceutical supplements in the thyroid setting: health benefits beyond basic nutrition. Nutrients. (2019) 11:2214. doi: 10.3390/nu11092214, PMID: 31540254 PMC6770945

[20] Sherrod LR Haggerty RJ Featherman DL. Introduction: Late adolescence and the transition to adulthood. J Res Adolesc. (1993) 3:217–26. doi: 10.1207/s15327795jra0303_1

[21] Aly H Raya Z Suzan G. Thyroid autoimmune disease among Emirati patients: A retrospective analysis of the patients’ characteristics. New Emirates Med J. (2024) 5:1–6. doi: 10.2174/0250688205666230904104404

[22] Abdulrahman AAA. The clinical significance of thyroid antibodies in non-thyroid diseases. World Fam Med J. (2023) 21:33–43. doi: 10.5742/MEWFM.2023.95256178

[23] Amouzegar A Gharibzadeh S Kazemian E Mehran L Tohidi M Azizi F. The prevalence, incidence and natural course of positive antithyroperoxidase antibodies in a population-based study: Tehran Thyroid Study. PLoS One. (2017) 12:e0169283. doi: 10.1371/journal.pone.0169283, PMID: 28052092 PMC5215694

[24] Kocełak P Owczarek AJ Wikarek A Ogarek N Oboza P Sieja M. Anti-thyroid antibodies in the relation to TSH levels and family history of thyroid diseases in young Caucasian women. Front Endocrinol. (2022) 13:1081157. doi: 10.3389/fendo.2022.1081157, PMID: 36605940 PMC9807877

[25] Dhillon-Smith RK Middleton LJ Sunner KK Cheed V Baker K Farrell-Carver S. Levothyroxine in women with thyroid peroxidase antibodies before conception. N Engl J Med. (2019) 380:1316–25. doi: 10.1056/NEJMoa1812537, PMID: 30907987

[26] Mammen JSR Cappola AR. Autoimmune thyroid disease in women. JAMA. (2021) 325:2392–3. doi: 10.1001/jama.2020.22196, PMID: 33938930 PMC10071442

[27] Wartofsky L. Management of subclinical hyperthyroidism. J Clin Endocrinol Metab. (2011) 96:59–61. doi: 10.1210/jc.2010-2409, PMID: 21209045

[28] Vanderpump MP. The epidemiology of thyroid disease. Br Med Bull. (2011) 99:39–51. doi: 10.1093/bmb/ldr030, PMID: 21893493

[29] Fröhlich E Wahl R. Thyroid autoimmunity: Role of anti-thyroid antibodies in thyroid and extra-thyroidal diseases. Front Immunol. (2017) 8:521. doi: 10.3389/fimmu.2017.00521, PMID: 28536577 PMC5422478

[30] Association of Clinical Biochemistry British Thyroid Association British Thyroid Foundation. UK Guidelines for the use of thyroid function tests. (2006), 1–86.

